# When Land Is Under Pressure Health Is Under Stress

**DOI:** 10.3390/ijerph18010136

**Published:** 2020-12-27

**Authors:** Aderita Sena, Kristie Ebi

**Affiliations:** 1 Centre of the Study and Research of Health Emergencies and Disasters, Oswaldo Cruz Foundation, Rio de Janeiro 21040-361, Brazil; 2 Department of Global Health, University of Washington, Seattle, Washington, WA 98195, USA; krisebi@uw.edu

**Keywords:** land degradation, desertification, drought, ecosystem services, sustainable development, climate change, human development, health

## Abstract

The land provides vital resources to support life on Earth. Land ecosystems services have social, cultural, and spiritual benefits and promote human health and well-being. However, human activities, particularly ongoing unsustainable land practices, are negatively impacting ecosystems through desertification, land degradation and drought (DLDD). This article highlights the pressures and impacts of DLDD on human health through exposure pathways, including water security and safety; sanitation and hygiene; food security and safety; air quality; and soil quality. We describe the impacts on 19 health outcomes in three groups: non-communicable diseases; injuries; and infections, parasitic and nutritional diseases. The magnitude of these health impacts is mediated by social, economic, and health system-related factors. We propose actions for the health sector to respond to the DLDD challenges.

## 1. Introduction

Land resources are vital for human health, well-being and, overall, life on earth. Land provides ecosystem services, as well as social, cultural, spiritual and economic benefits that form a life support system for human health and well-being [1,2]. Vital resources provided from ecosystems include food and essential nutrients; clean water and air; shelter; medicines and medicinal compounds; wood; fuel; fiber; energy; climatic constancy; regulation of risks of natural hazards and diseases; pollination; water purification; livelihoods; and cultural, spiritual and recreational enrichment [3]. Other benefits are related to biodiversity, which includes diversity within and among ecosystems and species that are essential to ecosystem functions and service delivery, as well as to the sustenance of human health and human well-being [4,5].

Human activities are negatively affecting ecosystem services and biodiversity through land degradation [2,6]. The drivers of land degradation and biodiversity loss are linked to population growth and rising urbanization, increased consumption, the expansion of crop and grazing lands, and unsustainable agricultural and forestry practices; these are within the context of unsustainable economic growth. In addition, climate change can affect the conditions of environmental and human systems, adversely impacting sustainable development [2,7,8].

The degradation of terrestrial and aquatic ecosystems is a problem of global dimensions. It affects every continent, from countries with large landmasses to small island states, from wet and dry regions to cold and warm ones, from wealthy developed countries to poorer developing countries. At least 3.2 billion people worldwide are affected by this complex phenomenon [8]. The most vulnerable and threatened land areas are the world’s drylands, although land degradation is also a large problem outside drylands [9].

As stated by the United Nations Convention to Combat Desertification (UNCCD), desertification means land degradation in arid, semiarid and dry sub-humid areas resulting from various factors, including climatic variations and human activities. Land degradation means reduction or loss, in arid, semiarid and dry sub-humid areas, of the biological or economic productivity and complexity of rain-fed cropland, irrigated cropland, or range, pasture, forest and woodlands resulting from land-uses or from a process or combination of processes, including processes arising from human activities and habitation patterns. Drought means the naturally occurring phenomenon that exists when precipitation has been significantly below normal recorded levels, causing serious hydrological imbalances that adversely affect land resource production systems [10]. This is known as meteorological drought. Other definitions specify the different types of drought, such as agricultural drought (when the shortage of precipitation impinges on crop growth due to soil moisture drought), hydrological drought (when surface and/or subsurface water supply is affected) [11,12,13], and socioeconomic drought (when the demand of commodities exceeds supplies due to water scarcity) [11,12]. There is general agreement that DLDD are challenges of a global dimension that continue to pose serious threats to the sustainable development of all countries, particularly of low- and middle-income countries, and specifically those in Africa [14].

DLDD challenges include health impacts. The links between DLDD and human health are complex. Most of the impacts are difficult to measure because they are indirect and mediated by global and local environmental, social and economic forces, such as climate change, level of deforestation, biodiversity loss, soil quality and erosion, economic activity, exploitation of natural resources, and other factors [15]. The negative impacts can be displaced across spatial and temporal scales [3,16]. Considering health impacts, damages from ecosystem disruption can follow complex pathways, with effects displaced geographically (e.g., health impacts observed in less developed countries, or the poor within one country, from overconsumption in wealthier regions) and into the future (e.g., health consequences of climate change and desertification for future generations) [3]. Similarly, DLDD poses multiple risks to livelihoods, and consequently, to human health [15,17]. DLDD reduces food production, freshwater access and ecosystem resources; as a result, health is placed under increasing stress.

One of the global responses is the UNCCD Strategic Framework for 2018–2030 that aims to restore the productivity of degraded land, and reduce the impacts of drought in affected areas, to achieve a land degradation-neutral world. This is consistent with the 2030 Agenda for Sustainable Development, specifically with Sustainable Development Goal (SDG) 15, “Life on Land”, and with the objectives of the UNCCD [18,19]. In addition, the goals and objectives of the UNCCD are in alignment with other two Rio Conventions—the Convention on Biological Diversity (CBD) and the United Nations Framework Convention on Climate Change (UNFCCC)—to address the complex challenges and interconnections between land, biodiversity and climate change [14,20].

Health is defined by the World Health Organization (WHO) as “a state of complete physical, mental and social well-being, and not merely the absence of disease or infirmity” [21]. Planetary Health, a recent concept, is defined as “the achievement of the highest attainable standard of health, well-being, and equity worldwide through judicious attention to the human systems—political, economic, and social—that shape the future of humanity and the earth’s natural systems that define the safe environmental limits within which humanity can flourish” [4,22]. Health also has been described as a “precondition for and an outcome and indicator of all three dimensions of sustainable development” [14]. A better understanding of the relationship between ecosystem sustainability and health benefits would be an important contribution to decision-making regarding health and environment management (including water, land, food, air, soil). This would ensure benefits to the health and well-being of all [3,15,23,24].

Although DLDD affects all regions, the negative impacts are disproportionately felt by people living in vulnerable conditions. These include poor women, indigenous communities, children, elderly persons, people living in rural, marginal or fragile environments, on land that is particularly vulnerable to degradation, as well as those with a low-income or living in poor areas [25,26,27,28]. This also applies to those without easy access to health care facilities and persons with pre-existing health conditions [26,29,30].

To respond to the concerns identified above, we conducted a targeted review to highlight the intersections between DLDD and health. The paper is organized into three parts. The first is the background highlighting the importance of healthy ecosystems to support life. The second describes the impacts on human health by key DLDD pathways (water security and safety, food security and safety, air quality, and soil quality) and by social, economic and health care system mediating factors that are determinants of health. The third is a discussion of social challenges (poverty and forced migration) and environmental challenges (lack of water security and lack of food security), and the directions to respond to the challenges to improve human livelihoods. Additional information can be found in the full report to the UNCCD on which this paper is based [31].

## 2. DLDD Pathways Affecting on Human Health

Land degradation can cause water and food insecurity, unemployment, gender inequality, conflict and migration. All ecosystem consequences from DLDD can affect human health and well-being, directly or indirectly, alone or combined [20]. Although land degradation is a major contributor to climate change [8], climate change also can aggravate these impacts, causing substantial costs in the environmental, social, economic and political dimensions, including in the health sector [20,32,33,34].

Climate change can exacerbate impacts on human health associated with DLDD (e.g., impacts from hot temperatures, from intense and prolonged extreme events such as drought and floods, and from declining freshwater resources and food security). Climate change accelerates soil erosion on degraded land through extreme weather events, which can increase the risk of forest fires. It can cause changes in the distribution of invasive species, vectors, pests and pathogens [8,33], influencing the occurrence of newly emerging diseases, such as zoonotic infections and vector-borne diseases, in areas without previous exposures [16,33,34,35,36,37]; and airborne pollutants can increase respiratory diseases [38].

Climate change is likely to contribute to changes in the geographical distribution of vector-borne diseases transmitted by mosquitoes (e.g., malaria, dengue fever, chikungunya fever, Zika, and Japanese encephalitis) and by ticks (tick-borne encephalitis and Lyme’s disease) due to changes in climate variables such as temperature, precipitation patterns, and humidity [34,39,40,41]. Likewise, modification of ecosystems due to anthropogenic activities such as deforestation, irrigation and water management may lead to the creation of mosquito breeding sites [3,42,43]. Drought-prone areas or dry conditions can also contribute to increasing this burden of disease by providing mosquitos with suitable breeding sites from unprotected water storage in households [36,44,45].

Extreme events such as droughts, floods, strong winds, dust storms, heatwaves and wildfires can cause diverse impacts on human health, including injuries, diseases and deaths. This can manifest in a number of ways, such as water-, vector- and food-borne diseases, air pollution, road injuries, falls, drowning, burns, physical traumas and venomous animal bites [16,46,47].

Moreover, when environmental vulnerability is placed in the context of social, economic and political challenges, including poor population health status, it can further increase the magnitude of all health impacts [26,29,33,48]. It is also worth noting that all populations are not equally vulnerable, and risks are not equally distributed [49,50]. All of these problems are likely to increase the threats to human health and quality of life [7,8].

**Figure 1:**
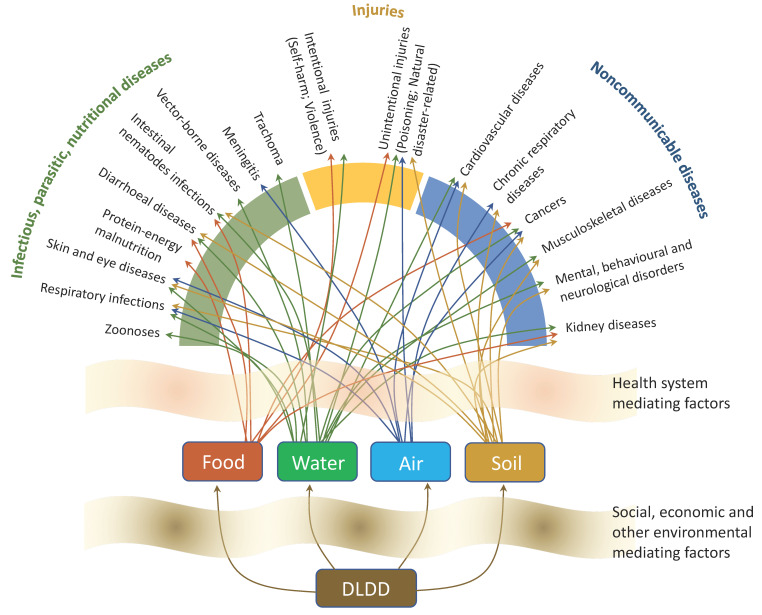
Desertification, land degradation and drought (DLDD) pathways and health effects; Source: [31].

Figure 1 shows the complex relationship between DLDD and health, following the WHO classification of major disease groups: Infectious, parasitic and nutritional diseases; Non-communicable diseases; and Injuries. Social, economic and environmental factors (which include water security and safety; sanitation and hygiene; food security and safety; air quality; and soil quality) act as mediating factors. Additionally, the quality of, and access to, health care services also act as mediating factors. Thus, DLDD related exposure pathways may result in 19 health outcomes.

### 2.1. Environmental Mediating Factors

#### 2.1.1. Water Security and Safety

Fresh water is essential for life and human health. Access is also a human right. The link between land management and the water cycle can determine the availability of water (quantity and quality). Land degradation practices can reduce water supplies, affecting agricultural systems and economic growth [8,51]. Climate and other drivers can make water resource-stressed conditions worse, such as population growth, economic development, urbanization, and land-use [52]. For example, in dry regions, more intense droughts will stress water supply systems, exacerbating water scarcity that, together with food shortages, can increase famine, which in turn can result in population migration [53].

Drought is an important factor in water security and health. It can impact the quality and quantity of safe water in several ways, including lack of management or mismanagement; contaminant concentration in ground and surface water; growth of pathogens from increased temperatures; high level of salinity in water; water stagnation due to reduced water level and stream flows; and damage of water-infrastructure supply [46,47,49,54]. Other important determinants of health related to water quality are industrial pollutants that can contaminate the water system. These include industrial chemicals, pharmaceuticals, and pesticides [24,55].

Lack of water quality and quantity can negatively affect human health with a wide range of consequences. Diseases linked with water pollution and water scarcity include infectious and parasitic diseases, non-communicable diseases, diseases associated with chemicals and other pollutants in water sources, including diseases related to algal blooms [46,47,49]. Water shortages can increase the operating costs of water services [32], affecting health services and people’s ability to buy water. It is also interrelated with negative socioeconomic dimensions, such as lack of or insufficient income, unemployment, or productivity loss [56,57,58,59]. These processes can increase mental health disorders, such as emotional stress, anxiety, and depression [49,58,59,60,61,62]. Other studies show evidence of mental health problems on farmers and farm workers when agricultural productivity and/or livestock were affected by the drought [57,63,64,65].

Lack of water may lead to disadvantaged persons having to carry water, increasing risks to musculoskeletal disorders [62,66]. It may also contribute to vector-borne diseases when people are forced to store water in unsafe containers creating mosquito breeding sites [36,44,45,47]. Esophageal cancer cases and mortality are suggested to be associated with high salinity levels of water, notably in regions or communities with water scarcity [67].

Lack of access to water, sanitation and hygiene (WASH) increase exposure to waterborne diseases, causing several infectious diseases, such as intestinal nematode diseases (worms), diarrheal diseases, cholera, skin (e.g., scabies) and eye infections (e.g., conjunctivitis, trachoma) [47,49,68,69,70,71,72]. Furthermore, the lack of access to WASH has an important role in undernutrition [73]; and can drive or maintain the cycle of poverty [74]. More than a third of the global population (about 2.4 billion people) still do not have access to sanitation facilities, and about one billion practice open defecation [75]. In 2016, 829,000 deaths globally from diarrheal diseases were attributable to unsafe drinking water, unsafe sanitation and lack of hygiene [76]. Diarrheal disease is the second leading cause of death in children under five years old worldwide, with 360 thousand out of 525 thousand being environment-related [73,77].

#### 2.1.2. Food Security and Safety

Productive terrestrial and aquatic ecosystems are the source of basic nutrition and energy, essential for health and overall well-being [3,20,78]. In many parts of the world, agricultural practices have become unsustainable because of increasing demographic pressures. These practices are depleting land resources, resulting in negative impacts on food security [79]. Lack of food security poses risks to economic status and human health, as well as to productivity (agricultural, fisheries and livestock) [20,29,79]. When ecosystems are affected, food supplies are reduced (essentially crops and livestock), which in turn reduce the quantity and quality of nutrient intake [49,79,80].

Lack of food availability can increase food prices, reducing access for people with low incomes or for those living in remote areas, impacting negatively on their nutritional status [54,81,82]. In rural and in poor areas, where people cannot purchase food, local food production is crucial to prevent hunger and to promote development and health [83]. Furthermore, the food–water nexus, if not secure, poses the risk of food shortages, and thereby also negatively affecting human health, economic growth and political stability [84]. Unsafe water and lack of hygiene in food preparation, inadequate food storage, and parasitic and chemical contaminants are all elements that jeopardize food safety [61,79].

Diarrheal diseases are the most common food- and waterborne bacterial disease outbreaks to occur in a prolonged dry season associated with dry conditions coupled with hot temperatures [85,86]. In addition, water and soil contamination can cause food-related exposures. Chemicals, such as cadmium, lead, arsenic, nickel, when consumed, can cause a range of health risks [87,88]. Cadmium can cause renal failure, osteoporosis and some types of cancers [89,90]; lead can cause increased high blood pressure and kidney damage, miscarriage, stillbirth, premature birth and low birth weight (in exposed pregnant women), and affect the development of the brain and nervous system in children [91]; arsenic can also cause renal failure leading to chronic kidney disease [88].

Droughts, floods, and warmer temperatures caused by climate change and variability can affect agriculture and fisheries, sometimes resulting in outbreaks of food-borne illnesses [33,71,92]. These risk factors also affect crop productivity, increasing the risk of food shortages, thus increasing the risk of undernutrition, especially in low-income countries [33,93]. Drought is a major driver of food insecurity [48,92]. As an example, drought impacts affected approximately 150 million people and caused USD 23.5 billion worth of losses on crop and livestock production in sub-Saharan Africa, which represents almost 77 percent of all production losses worldwide (based on data from 2003 to 2013) [80].

Lack of quality foods may result in reduced quantity and/or quality of nutrient intake, increasing malnutrition prevalence (in all its forms) and mortality risks [49,69,78,92,94]. Undernutrition can be chronic, leading to stunting (low height for age), with 144 million affected children under 5 years of age globally. Acute undernutrition can cause wasting (low weight for height), affecting 47 million children under 5 globally [95]. Both chronic and acute undernutrition increase childhood mortality, particularly from comorbidity with infectious diseases, such as pneumonia and diarrhea. A vicious cycle exists between undernutrition and infectious diseases [47,72,94]. Micronutrient deficiencies can additionally result in secondary health outcomes, such as anemia from lack of iron, eye problems, in particular, night blindness from vitamin A deficiency, and scurvy from vitamin C deficiency [29,49,96]. Micronutrient reduction coupled with undernutrition in low- and middle-income countries is linked with lost pregnancies, premature births, fetal growth reduction, neonatal and child deaths, and contribute to lower development of children’s cognitive potential [83,97,98,99]. A different form of malnutrition leads to overweight caused by consuming low-quality, high-calorie food, which contribute to non-communicable diseases in adult life [79,96]. Globally, an estimated 38 million children under five years of age are overweight [95].

Food production also has health impacts. For example, studies have confirmed an increasing number of patients with chronic kidney diseases in rural agricultural communities. Agricultural workers are in particular at risk to develop chronic kidney disease due to their large and frequent exposure to several agrochemicals, heavy metals, heat stress and dehydration; even those who are not agricultural workers but live in communities with high agricultural activity are adversely affected [55,100].

#### 2.1.3. Air Quality

Dry and dusty conditions, fires from land clearing, wildfires, dust storms are all causes of impaired air quality. The general effects of air pollution on morbidity and mortality are: (a) premature deaths due to cardiovascular and respiratory diseases, lung cancer, and acute lower respiratory infectious (e.g., pneumonia); (b) irritation of the respiratory tract, causing respiratory disorders (e.g., asthma, tracheitis, pneumonia, allergic rhinitis, desert lung syndrome); and (c) causing or aggravating bronchitis, emphysema, cardiovascular diseases (e.g., hypertension, stroke, increasing the risk for acute myocardial infarction, inducing atherosclerosis), eye infection, skin irritations, and meningococcal meningitis. Dust is also related to deaths and injuries due to reduced visibility, which can cause road accidents [16,24,101,102] and poses risks for aviation traffic [101,102].

Problems associated with dust storms can be intensified by degradation in drylands [102,103]. The physical, chemical, and biological properties of airborne dust pollution (including mineral dust and dust storms exposure) and other pollutants pose risks to human health [69,102]. Dust can be harmful through pathogen carriage [104] and direct trauma by inhalation of particulates. Hazardous dust particles include fine mineral particulates and a combination of pollutants, spores, bacteria, fungi and potential allergens that are carried along with mineral dusts [101,102]. Mineral dusts can cause some types of cancers (e.g., liver, kidney) and other serious diseases, such as renal failure and osteoporosis [90]. Some allergic respiratory diseases are climate-sensitive. In warmer conditions coupled with situations of drought and wind-borne dust, airborne allergens (fungal spores and plant pollen) are produced and released, causing asthma and allergic rhinitis [38,102,105]. Furthermore, inhalation of fungal spores carried in air dust may result in outbreaks of Valley fever (caused by a fungus—coccidioidomycosis), for instance, in drylands areas, such as Southwest US, Northern Mexico, and Northeast Brazil [101,102].

Wildfires increase the risk of unintentional injuries and deaths from extreme heat, burns and smoke inhalation [16,38,106,107]. Animal bites may occur in cases of deforestation, floods, storms, drought events, water and food shortage, and excessively hot weather when animals move their habitat to areas where humans live [73].

Furthermore, wildfires caused by heatwaves, drought and increased soil erosion can affect large numbers of people for days to months due to exposures to particulate matter and other toxic substances, including burns and smoke inhalation [107,108,109]. As an example, worldwide, an estimated 339,000 (range 260,000 to 600,000) premature deaths occur annually from air pollution from forest fires, with sub-Saharan Africa and Southeast Asia the most-affected regions [110]. Additionally, high temperatures can move volatile and semi-volatile compounds from water and wastewater into the atmosphere, which, in turn, change the distribution of contaminants, increasing people’s exposure [55,111,112].

In a recent study, Landrigan et al. [24] identified emerging evidence of an additional causal association between fine particulate matter (PM_2.5_) pollution and some non-communicable diseases, such as diabetes, attention-deficit or hyperactivity disorders in children, decreased cognition function, the occurrence of neurodegenerative disease (e.g., dementia) in adults, as well as increased premature birth and occurrences of low birth weight. Other studies indicate that dry seasons, coupled with low humidity and high airborne dust concentrations, may result in meningococcal meningitis outbreaks with high fatality rates, specifically in Africa in a semiarid region known as “the meningitis belt” [20,102,113,114].

The most vulnerable populations exposed to dust are in arid and adjacent areas, such as the Middle East, North Africa, the Sahel, Australia, China, the US Southwest, and Mexico; although exposure can affect populations far from these regions (e.g., dust transported from China and Mongolia to Japan and Korea) [101,102]. In some parts of the world, dust storm frequency is changing due to land-use and climatic change [101]. People who suffer the greatest impacts are children, the elderly, and especially people with chronic health conditions like respiratory and heart conditions and lung diseases, and those in high exposure situations (e.g., agricultural or outdoor laborers; and people living close to desert areas or industries) [24,54,102]. For instance, dust from the Chihuahuan Desert has led to increased hospital admissions for children (aged 1–17) due to asthma and bronchitis in El Paso, Texas. The same study also found that girls are more sensitive to acute bronchitis hospitalizations after dust events than boys [115]. Respiratory mortality among the elderly in Italy (aged 75 or older) and Spain increased during Saharan dust events [116,117].

#### 2.1.4. Soil Quality

Soil is an essential resource for ecosystem functions and is responsible for 95 percent of food production. The soil has an important function in filtering naturally existing contaminants. These compounds are mainly formed through soil microbial activity and decomposition of organisms (e.g., plants and animals) [118,119]. Soil contamination, degradation and erosion are reducing the productivity of agriculture and livestock in many areas of the world, affecting food security and human health [120,121].

Soil degradation and pollution can result from poor agricultural practices, high levels of chemical elements (native or introduced) or hazardous substances from industrial, military and extractive activities, inadequate irrigation process, improper solid waste management (including hazardous nuclear waste and unsafe chemical storage) [87,119,120,122]. When soil is depleted, the filter function can fail, and contaminants transferred to water systems and the food chain [119,121]. Some heavy metals such as mercury, lead, arsenic, cadmium and chromium, coupled with pesticide pollutants and pharmaceuticals used for livestock management (e.g., antibiotics), are degrading soil biodiversity and their function. This situation, in turn, poses risks to agricultural productivity, livelihoods, food security and human health, as well as to wildlife [87,112].

Unintentional poisoning can be associated with environmental contamination from climate, behavioral, agricultural and developmental pathways. Soil contaminated by the constant use of pesticides, airborne toxins from contaminated dust, water and soil (e.g., algal bloom cyanobacteria), use of chemical products, and air pollution by chemicals from industries or some occupational activities are all risk factors for the poisoning of ecosystems and people [67,123]. United Nations Environment Program (UNEP) estimates that every year 25 million agricultural workers worldwide experience unintentional pesticide poisoning [87].

Contaminated soil can affect human health through three main routes: inhalation, ingestion and the skin. The effects on human health include increased risk of cancer; harmful effects on the nervous, digestive and immune systems, lungs and kidney; skeletal and bone diseases; sterility and other reproductive disorders; immunity suppression; neurological development damage and low IQ; and increased antimicrobial resistance [87,112,122]. Lymphoma, multiple myelomas and leukemia have several causal links with pesticides and herbicides used in agricultural practices accounting for an important fraction [112]. Likewise, arsenic (with potential sources, including food contaminated by pesticides, seafood, and groundwater) can cause renal failure leading to chronic kidney disease [88].

Examples of health impacts associated with DLDD drivers are summarized in Table 1.

### 2.2. Social, Economic and Health System Mediating Factors

The causal chain from DLDD through food, water, air and soil quality is mediated by social, economic and other environmental factors and by the response of the health system. Social and economic factors can contribute to vulnerabilities at the local level, especially in poor communities and in cases where the impacts are of long duration. However, these factors are also mediated by other local forces, such as other non-DLDD environmental risks, and by local cultural and political factors [7,13,124,125].

The susceptibility of poorer countries and regions to DLDD can be made worse by direct impacts from climate extremes and climate variability [7,50,126] and by the lack of access to public health care services [29,127]. Damage to, or inadequate physical conditions of, infrastructure that supports human livelihoods (e.g., supply of power and water for drinking and hygiene, waste management, and sanitation), as well as reduced food security and access to health care, can increase health risks [7,13,33,62,128]. For example, in Cuba, where the public health system is well developed, the lack of drinking-water supply in some communities led to inadequate storage practices by the population, contributing to persistent dengue fever cases [129].

In rural communities that are agriculture-dependent, especially in a low-income context, drought and land degradation can exacerbate forced migration, violence and conflict due to food insecurity and can thus cause social instability and mental health [59,60,83].

Factors that influence vulnerability include race, gender, ethnicity, poverty, social inequalities and culture, all of which can impair health status and increase social disadvantage [13,33,130,131]. For instance, some indigenous communities have higher risks of economic losses and poor health if they live in areas vulnerable to climate change and if they are dependent on local natural resources [33,132]. For women and children, lower health status can be related to low educational level, low socioeconomic status, low perception of the illness’ seriousness, and cultural behaviors [29,133]. In disaster situations, gender is a strong factor that increases vulnerability. The expected roles and relations between men and women in a given culture can determine gender differences, including norms and values, that in turn can increase gender inequalities [134,135].

Health services can reduce the health impacts of DLDD, while the health system itself can be affected by DLDD. Lack of water, water shortages and contaminated water can pose risks to some basic health care and hospital procedures (e.g., vaccine application, dressing wounds, surgeries) and worsened working conditions, which may, in turn, affect the health conditions of the population [29,62]. Simultaneously, the increasing impacts on human health arising from risk factors related to DLDD require more health care services and the costs of this increase [33,49].

Access to health services can often be limited, especially in low- and middle-income countries, among poor people and among those who suffer a disproportionally high burden of disease [29]. Migration and family disruption can also increase health problems, including mental health, and create other family and social changes; this also predominantly affects poor people, who do not have the necessary financial conditions to receive adequate health care [29,49,62]. Empowerment of people is important because it provides choices for protecting their health (e.g., healthy food, healthy lifestyles) as well as the knowledge of when health services are need [127].

Table 2 summarizes some examples of social, economic, and health care mediating factors that can affect human health.

## 3. Discussion: Challenges and Directions to Improve Human Livelihoods

Complex and intermingled environmental and social challenges of DLDD affect communities and population health. Four of the many challenges were highlighted in the UNCCD Strategic Framework 2018–2030: poverty and forced migration (key social challenges); and lack of water security and lack of food security (key environmental challenges).

### 3.1. Poverty

Lower-income groups are more dependent on the agricultural sector, as compared to the general population, and have access to land with lower productivity, intensifying poverty and income inequality [6,33,138]. The outcome of the analysis in selected low- and middle-income countries showed that people living on fragile lands presented a higher overall proportion of rural poverty [27]. For instance, 50 percent of the total population of sub-Saharan Africa lives in drylands, with 75 percent of the population in those areas living in poverty [8].

By 2050, four billion people are projected to live in dryland areas with decreased land productivity. This, coupled with poverty and other social stresses, can make people more vulnerable to socioeconomic instability and violent conflict [138]. Poverty and lack of access to health care form a complex vicious cycle. Poverty leads to ill health that can maintain poverty [127,131]. Although access to health care services is improving in low- and middle-income countries, there are large differences in the equity of access. For example, poor people have less or limited access to services in some settings, which in turn may result in an increased burden of disease [29,127]. Poverty and gender inequality are associated with lower levels of power and access to choices and resources [135]. Poverty reduction goes hand-in-hand with declining vulnerability [139].

### 3.2. Forced Migration

DLDD has a complex and frequent association with population migration. Annually, tens of millions of people, many living in rural areas of low- and middle-income countries, migrate for reasons related to land degradation (e.g., food scarcity), extreme weather and climate events (e.g., drought, floods), and the effects of climate change on food security [78]. Depletion of natural resources due to environmental degradation also poses risks for rural people’s subsistence, especially those depending on agricultural production, livestock, fisheries and forest-based livelihoods. Intervening mediating factors include social, economic, political infrastructure, and demographic factors [53,138].

International migration occurs mainly from low- and middle-income to high-income countries. However, most migration takes place within national boundaries or between contiguous countries. The most common internal migration pattern occurs from rural to urban areas, and also within rural areas and between cities (inter-urban migration). The temporal dimensions vary, which can be temporary or permanent [53]. It is estimated that globally by 2050, between 150 and 200 million people could be displaced for environmental reasons, including desertification, land degradation, sea-level rise and increased extreme weather events [51,140]. Some estimates are as high as 700 million [8,141].

People who migrate can face many challenges at different stages of migration. They may experience violence and discrimination in the places where they transit through or migrate to [140]. There are challenges faced by families left behind, especially if they do not have the ability to cope with economic, social, environmental, political and security vulnerabilities [29,46,142,143]. Socioeconomic inequality is a key factor in driving the migration process in areas with land degradation; the degree of vulnerability is amplified when coupled with social injustice and unsustainable development [78,120]. In addition, in drylands, it is likely that hunger and poverty-related vulnerabilities will increase, potentially resulting in a cycle of the perpetuation of poverty [13,60,130,131,138].

All types of migration can affect family structures and dynamics. Changes in household dynamics can have negative effects on family health and well-being [144]. Family separation can cause negative effects on mental health and behavioral disorders, can heighten the risk of infectious diseases and can worsen pre-existing health conditions (e.g., cardiovascular diseases) [17,49,62]. Displaced people can experience changes to their routine hygiene behavior, leading to a relaxing of practices that avoid infectious diseases and/or promote health [36,46]. On the other hand, migration (seasonal or long-term) can provide basic subsistence for family members, such as food and water security and nutrition [144].

### 3.3. Water Security

Current levels of water withdrawals are not sustainable. Water demand between 2015 and 2030 is expected to increase by 30 percent, a situation that is already leading to conflicts in some parts of the world [145,146]. Climate change is an important additional driver of water scarcity. There is robust evidence as to its role in the reduction of renewable surface water and groundwater resources in most dry subtropical regions in the near future. Greater variability of precipitation can cause short-term shortages due to increased runoff in some water-stressed areas, reduced seasonal distribution of change in streamflow, and reduced snow and ice storage. In addition, the availability of drinking water can be impaired by the presence of algae-producing toxins caused by high temperature and by pathogens and pollutants [47,51,52,84]. This will have clear implications for agriculture and livestock, and therefore for food security, as multiple sectors compete for water resources [52]. Population health can suffer if health services are affected by a lack of water security and water quality. This can occur due to deficiencies in health care procedures and worsening health working conditions [29,47,60].

Droughts are likely to intensify in already affected areas in southern Europe and the Mediterranean region, central Europe, central and southern North America, Central America, northeast Brazil, and southern Africa [52]. There is a medium- to near-term (2030–2040) risk of significantly reduced renewable water resources in most dry subtropical regions, but adaptation mechanisms can reduce the risk to the category of low-risk. An increase of 2 °C in the long term (2080–2100) could result in high-risk, with adaptation measures reducing the risk category to medium [52].

### 3.4. Food Security

One of humanity’s greatest challenges is to ensure healthy diets for a growing world population while ensuring healthy and sustainable food systems. Globally, more than 820 million people have insufficient food, leading to malnutrition and the risk of infectious diseases. An even larger number of people consume an unhealthy diet that contributes to premature death and morbidity from non-communicable diseases. These extremes occur while pressures on food systems increase [147]. As populations increase and standards of living and nutrition improve, the demand for food will continue to rise.

The current world population is 7.7 billion and is projected to increase to 8.5 billion in 2030, to 9.7 billion in 2050, and higher towards the end of the century [148], with much of the rate of growth in low-income countries [149]. This growth will add further pressure on land-based and aquatic food-producing systems [150]. The challenge is how to produce more food, of better nutritional quality, for an increasing population but without further stressing the land.

There is a link between disasters and food security. A review of 78 post-disaster assessments (including droughts, floods, tropical storms and forest fires) from 48 low- and middle-income countries (in Asia, Africa and Latin America) over 2003–2013 showed that the largest impacts occurred in low- and middle-income countries, particularly in the agricultural sector. Of the post-disaster economic impact of USD 140 billion, 22 percent was related to the agriculture sector (with 42 percent of damage and loss in crop production) and 36 percent to livestock, with 44 percent caused by drought events and 39 percent by floods, with major impacts particularly in Asia and Africa. Similarly, the indirect impact of disasters such as forest fires was a major risk for people whose livelihoods are linked to forests [80].

### 3.5. Responding to the Challenges

The UNCCD places “humans at the center of concerns to combat desertification and to mitigate the effects of drought” [10]; and the second and third objectives of the UNCCD Strategic Framework for 2018–2030 aim, respectively, to “improve the living conditions of affected populations”, and to “mitigate, adapt to, and manage the effects of drought in order to enhance the resilience of vulnerable populations and ecosystems” [18]. The United Nations Conference on Sustainable Development (Rio+20) also recognized that to achieve the goals of sustainable development; this is necessary to work to reduce the high prevalence of debilitating communicable and non-communicable diseases and to ensure populations are able to reach a state of physical, mental and social well-being [14]. A sustainable and equitable development regarding land and water management, combined with measures of climate change mitigation and adaptation at local and global levels, will facilitate the achievement of the SDGs, especially in order to improve nutrition and human health and reduce poverty [15,120].

Interagency and intersectoral action are critical to achieving the 2030 Agenda for Sustainable Development. For DLDD, this means to specifically address SDG15 “*Protect, restore and promote sustainable use of terrestrial ecosystems, sustainably manage forests, combat desertification, and halt and reverse land degradation and halt biodiversity loss*” and target 15.3, which states: “*by 2030, combat desertification, restore degraded land and soil, including land affected by desertification, drought and floods, and strive to achieve a land degradation-neutral world*”. Implementing actions based on SDG 15 at the national and local levels, based on international agreements, is essential for development without land degradation and biodiversity loss, therefore supporting health and well-being [8]. Regarding SDG3, “*Ensure healthy lives and promote well-being for all at all ages*”, health and well-being depend on the sustainable management of natural resources. Thus, healthy ecosystems are needed to ensure healthy communities and societies. Land degradation neutrality will support nutrition, and long-term water security, food security, as well as poverty-reduction [8,15]. Progress towards SDG 3 and SDG 15 will also contribute to accomplishing other SDGs.

Successful strategies based on local, sustainable land management have a significant potential for upscaling [1]. Timely and appropriate actions in avoiding, reducing and reversing land degradation are necessary to obtain multiple benefits, including providing food and water security; contributing to adaptation and mitigation measures for climate change; decreasing disaster risks; protecting human health; increasing socioeconomic stability; and avoiding or reducing conflict and migration [8,151,152].

Nonetheless, DLDD impacts on human health and well-being are often not well recognized by the health sector or even by other sectors and stakeholder groups. Thus, strengthening health systems to provide equitable and universal health coverage must include an integrative approach with DLDD interventions [14,153]. Thus, the health sector should be included in the decision-making process in all policies [154]. Recommended strategies include increased government awareness and political commitments to support coordinated and integrated policy agendas [139,155] and established financial mechanisms to support social protection policies to enhance life-saving and productive safety-nets [155,156,157,158].

In addition, developing pro-active approaches with community participation to reduce vulnerabilities and improve preventive and response capacities of people at risk is a key measure to avoid health impacts and promote health [60,146,157,159]. Similarly, there are opportunities to support local systems and to implement resilient policies that respond to the DLDD challenges affecting health [1,2,50].

Table 3 provides examples of health sector actions to address the new realities emerging from DLDD.

## 4. Conclusions

The current state of DLDD calls for urgent actions to protect human health and well-being. Lack of awareness of DLDD and its drivers and impacts are a barrier to action. Raising knowledge and awareness of the DLDD driving forces and consequences in the social, economic and environmental dimensions is crucial and should be implemented at every level. Actions across jurisdictions and sectors are needed at every stage of the pathways from DLDD drivers, their exposures, and their human health impacts [20,162]. Certainly, actions at the highest possible level (drivers) are more effective than actions taken when the health impacts have already occurred (curative actions). Therefore, the protection of essential ecosystem services must be ensured and sustained.

Responding to DLDD challenges will need a global effort. Governments, communities, civil society and the private sector play important roles in DLDD and in responding to current and emerging environmental challenges. Simultaneously, the social determinants of health need to be addressed, such as poverty reduction, water and food security, livelihood support, capacity building, and overall building resilience and human development.

## Figures and Tables

**Table 1:** DLDD drivers affecting human health through water security and safety, sanitation and hygiene, food security and safety, and air and soil quality.

DLDD Drivers	Environmental and Social Pathways	Human Health Impacts (Morbidity and Mortality)
Water Security and Safety	Water shortage Consequences of water quality (non-potable water, saline water) Contamination of water by various means, such as toxic algal blooms, bacteria, fungi, virus, toxins, chemical pollutants Damages to health services functioning, with consequences to the provision of some sanitary procedures Consequences on the water supply and distribution system (for piped water, water trucks, cisterns, artesian wells, dams and other alternative sources) Household water collection and storage, which may compromise water quality Water collection and transport (which may cause physical injuries) Change in vectors, hosts and reservoir cycles Effect on irrigation for agricultural production and in livestock and fishing increasing the possibility of food shortages Impaired hygiene (personal, household, food, health service equipment) due to lack of water Consequences of sanitation services, urban cleaning, health services and other basic services	Gastrointestinal infectious diseases (diarrhea, hepatitis A, typhoid fever, and other infections) Parasitic infections (intestinal nematodes infections) Dermatological infectious diseases (scabies) Diseases transmitted by vectors and zoonoses (e.g., dengue, Zika, chikungunya, malaria, leishmaniasis, leptospirosis) Infectious diseases transmitted by viruses, bacteria, fungi (flu, pneumonia, conjunctivitis, trachoma, scabies, and other diseases) Cardiovascular diseases (e.g., hypertension) Kidney diseases Cancers (esophageal cancer) Dehydration Undernutrition Unintentional injuries (poisoning by toxins) Musculoskeletal disorders (bone damage, back and muscle pain) Mental and behavioral disorders (stress, anxiety, depression)
Food Security and Safety	Deficiency in agricultural, livestock and fishery production causing food shortages Difficulty in the sustainability of family agriculture, livestock and fishery Food contamination (microbiological and chemical) Rising food prices Decreased access to food, especially to healthy food	Nutritional deficiencies (anemia, night blindness, scurvy) Malnutrition and its complications (low physical and cognitive development, deficiency of the immune system, overweight) Fetal growth restriction, neonatal and child deaths Infections from food contaminated by viruses, bacteria, fungi, parasites (diarrhea, cholera, hepatitis A, worms, other infections) Chronic diseases (hypertension, obesity, cancers, diabetes) Renal and kidney damages Mental, behavioral and neurological disorders (stress, anxiety, depression, suicide) Unintentional injuries (poisoning)
Air Quality	Low humidity Increased temperature (heat, warmer conditions) Dust storms, dust particles Air contamination by particles from fires (wildfires, agricultural practices) and toxins accumulated in air, soil and water Accidents caused by reduced visibility Release of airborne allergens (fungal spores and plant pollen)	Acute respiratory diseases (flu, sinusitis, rhinitis, bronchitis, pneumonia) Chronic respiratory diseases (asthma, allergic rhinitis, chronic obstructive pulmonary disease) Cardiovascular diseases (stroke, ischemic heart disease, hypertensive heart disease) Cancer (lung, bronchus, trachea, liver, kidney) Neurodegenerative disorders Skin irritations (dermatitis) and eye infection (conjunctivitis) Meningococcal meningitis Diseases caused by fungi, viruses, algae, bacteria, allergens Valley fever Premature births and low birth weight Unintentional injuries by road accidents
Soil Quality	Loss of productive soil leads to lower food production, from decreasing agricultural yields and livestock, causing food shortages Soil contamination from chemical products Soil contamination from animal and human excreta Air contamination through contaminated dust	Infections from food contaminated by viruses, bacteria, fungi, parasites (diarrhea, cholera, hepatitis A, worms, other infections) Non-communicable diseases (types of cancers, neurological damage, lung and kidney diseases, skeletal and bone diseases, sterility and reproductive disorders, immune suppression) Respiratory infections (e.g., pneumonia) Skin and eye irritation and allergies or infections Nutritional deficiencies Unintentional injuries (poisonings)

Source: [16,20,24,29,33,45,46,47,49,59,61,62,66,67,69,87,96,101,102,112,122]. Note: each pathway can have multiple health impacts (not shown).

**Table 2:** Pathways and main health impacts from social, economic and health care system mediating factors.

Mediating Factors	Environmental and Social Pathways	Human Health Impacts (Morbidity and Mortality)
Social and Economic Factors	Loss and damage of livestock and subsistence plantations due to difficulty in accessing water Loss or lack of employment, or low income Social impacts from the need to collect water (gender differences, opportunity loss) Lack of access to drinking-water leading to inadequate water storage, and use of contaminated water Lack of hygiene conditions and practices Lack of access to food and the ability of sustenance Rising water prices due to scarcity and high purchase demand Migration of populations due to conflict or seeking improvement in their quality of life (resulting in other social and cultural changes and changes in the epidemiological profile of migrants and of the receiving areas) Family separation (displacement of a family member to other areas in search of employment to supply family needs) causing disruption and changes in the family structure and dynamics Loss of social identity; uncertainty and concerns for the future; lower levels of hopefulness and of social support	Psychological disorders (anxiety, stress, behavioral change generating other problems such as violence, alcoholism, depression) Suicide Increasing chronic non-communicable diseases (cardiovascular diseases, back and arms pain) Increasing infectious and parasitic diseases (gastrointestinal diseases, worms, water and vector-borne diseases) Increasing demand for health services and other social problems in the places where people migrate to
Health System Factors	Risks of interruption of health care procedures due to lack of water Increased demands for care and supply of health services Risk of impacts in energy supply, impairing the use of health equipment, refrigeration of medicines and vaccines and other medical supplies, and the health care of some facilities services	Increasing communicable and non-communicable diseases Lack of, or reduction of, health care due to lack of working conditions that may worsen the health conditions of the population

Source: [26,29,30,33,36,47,49,59,60,61,62,131,136,137]. Note: each pathway can have multiple health impacts (not shown).

**Table 3:** Health sector actions are needed to respond to DLDD challenges.

**Adaptation/Coordination**
Ensure that the health sector works across sectors and in coordination with national policies so as to address and respond to drivers of DLDD, rather than focusing only on reactive responses in the form of curative services. An intra and inter-sectoral dialog to discuss DLDD drivers and impacts on health is needed. Adapt public health systems to the realities of local ecosystems. This is key to improve human health and well-being and avoid or reduce future impacts.
**Strengthening Capacity-Building and Resilience**
Ensure preparedness of public health agencies to respond to the health impacts of DLDD with the affected communities; strengthen capacity-building for health professionals and communities, according to local DLDD realities, in order to increase resilience. Promote access to prevention, treatment, care and support with regard to all DLDD impacts on health, such as infectious diseases, non-communicable diseases, and injuries.
**Emergency Preparedness and Response to Disasters**
Develop (or strengthen) strategic plans for emergency preparedness and response to disasters (e.g., drought, disease outbreaks, landslides, dust storms, tropical storms), considering the local infrastructure and political context. Implement early warning systems to reduce vulnerability and improve preventive and response capacities of people at risk.
**Monitoring and Surveillance**
Strengthen (or develop) disease monitoring and surveillance systems (both infectious and non-communicable diseases, including mental health and malnutrition) to detect and control DLDD related diseases with the integration of approaches among key sectors. Implement a systematic program of surveillance and analysis of drinking-water between water agencies and health sectors so as to ensure water quality according to a national drinking water quality standard. Establish a mechanism to integrate climate services into health surveillance policy in order to monitor meteorological forecasts and climate-sensitive diseases.
**Assessment**
Implement a mechanism for mapping and assessing local DLDD risks, including vulnerabilities, hazards and exposures (population and health infrastructure) to identify particular and effective adaptation measures. Assess the risk posed by toxic chemicals and pathogens from agricultural practices.
**Education and Communication**
Implement education and communication strategies in urban, rural, poor and remote areas to increase community awareness regarding DLDD health risks related to water security; safe water supply and sanitation; source water protection; household water treatment, storage and reuse; food security and information on nutrition; mechanisms to reduce air pollution, including dust; and use of agricultural chemicals. Implement health education and promotion strategies to increase community awareness about risk factors for human health, considering gender differences, educational gaps, socioeconomic status and cultural behaviors.

Source based on [4,13,14,16,29,30,35,47,48,50,52,54,137,139,152,157,159,160,161].

## Data Availability

The data presented in this study are available within the article.
